# Electrochemical Coupled Analysis of a Micro Piezo-Driven Focusing Mechanism

**DOI:** 10.3390/mi11020216

**Published:** 2020-02-20

**Authors:** Chong Li, Kang Liang, Wei Zhong, Jiwen Fang, Lining Sun, Yong Zhu

**Affiliations:** 1 School of Mechanical Engineering, Jiangsu University of Science and Technology, Zhenjiang 212003, China; 199020032@stu.just.edu.cn (K.L.); zhongwei@just.edu.cn (W.Z.); fjw617@just.edu.cn (J.F.); 2 Robotics and Microsystems Center, Scoochow University, Suzhou 215021, China; lnsun@hit.edu.cn; 3 Suzhou Mingzhi Technology Co., Ltd., Suzhou 215217, China; 4 National Research Center of Pumps, Jiangsu University, Zhenjiang 212003, China

**Keywords:** numerical analysis, electrochemical coupled, piezo-driven, focusing mechanism

## Abstract

In order to improve the response speed and output force of the camera focusing mechanism, the authors proposed a novelty micro focusing mechanism based on piezoelectric driving, which has the characteristics of rapid response, high precision positioning and large displacement focusing. In this paper, the operating principle of the proposed focusing mechanism is presented. Using the piezoelectric output characteristic, the movable tooth drive theory and the screw drive theory, the electromechanical coupling mechanical model and equations of the piezoelectric focusing mechanism are established. Through MATLAB simulation, the output characteristics of the piezoelectric focusing mechanism are calculated. The results indicate that the maximum thrust force of the lens and the maximum output torque of the movable tooth drive for the piezoelectric focusing mechanism are 562.5 N and 1.16 Nm, respectively. Furthermore, the driving voltage directly affects the output performance of the piezoelectric focusing mechanism. These results can be utilized both to optimize the dimensions and improve the overall performance of the piezo-driven focusing mechanism.

## 1. Introduction

The rapid development of intelligent manufacturing has propelled electromechanical and microelectromechanical systems to pursue high precision, integration and intelligence [1,2,3,4]. At the same time, higher requirements of anti-interference, anti-impact and anti-vibration are put forward for various precise transmission devices and mechanisms [5,6,7]. Focusing mechanism is an electromechanical drive system used to adjust the focal length of a camera. As the focal length of the lens changes, the scope of the target to be photographed varies, and the effect of the photo is quite different. Hence, the accuracy and quick response characteristics of the focusing mechanism have a significant influence on the performance of the camera [8,9].

Piezoelectric element has many excellent performances, such as fast response, small size, large output force and no electromagnetic interference. As a result, it has been successfully used in the field of robot actuating, precision positioning, energy harvesting and vibration reduction [10,11,12,13]. 

The driving of the camera focusing mechanism is an important application of piezoelectric actuation in the precise positioning field. Numerous achievements on improving the performance of the focusing system have been done by researchers. As early as 1987, Canon Company applied its independently developed annular piezoelectric ultrasonic motor to the focusing system of the EOS camera. The application of piezoelectric driving not only enables the camera to focus quickly, but also simplifies the camera structure and reduces its weight. Currently, Canon has installed piezoelectric motors in more than 30 types of cameras [14]. Burhanettin et al. [15], scholars from Pennsylvania State University, developed a single-phase piezoelectric ultrasonic motor driven by the bending vibration mode of metal cylinders, which was successfully applied to the focusing system of mobile phone cameras by Samsung Company. Subsequently, Sigma, Nikon, Pantex and other companies also use piezoelectric drive to achieve camera focusing. In recent years, the study of using piezoelectric to drive the various optical lenses focusing mechanism has gradually increased. Japanese researcher Masahiko et al. [16] applied a spherical piezoelectric ultrasonic motor to achieve the positioning and driving of the camera inside the tube. With this driving method, the camera can rotate 360 degrees along the tube. With eight piezoelectric films, Australian Michael et al. [17] designed an actuator used for out-of-plane movement of the microlens. Compared with other lens-driven mechanisms, this mechanism can achieve higher response frequency and is suitable for rapid action. Based on piezoelectric driving, Nazmul et al. [18] in US designed a kind of adjustable focusing adaptive glasses, which has practical value for improving the visual degradation of people. According to the characteristics of high controllability and automatic focusing required by mobile phone cameras, Korean Hyun-Phill et al. [19] developed a piezoelectric actuator for automatic focusing of mobile phone cameras. When focusing, the response time of the piezoelectric actuator is only 1% of the automatic focusing time, and it consumes energy only when working. The average instantaneous power is only 65 mW, which is far higher than the traditional electromagnetic mechanism in terms of energy conversion efficiency. What is more, since in the laser direct writing equipment the focusing mechanism with high resolution is needed to focus the photoresist objective lens, Chinese researcher Liu et al. [20] developed a focusing servo mechanism for laser direct writing equipment based on piezoelectric driving. As the proposed focusing mechanism has the characteristics of 10 nm resolution, 10 μm travel and 1.37" guiding accuracy, so it is an ideal focusing mechanism that is suitable for nanometer positioning. Chen et al. [21] developed a piezoelectric screw micromotor for the automatic focusing and zooming of the micro camera. The system uses the hollow piezoelectric stator as the nut stator, and the hollow rotor is connected with the lens as the stud to generate the spiral motion along the axial direction. 

For the piezoelectric-driven focusing system, the performance of the control system is one of the key factors that affects its response speed and stability. Therefore, scholars have done a lot of research on improving the control performance of the focusing system. Teo et al. [22] constructed a PI tracking controller based on the integral force feedback, and verified the control effect of the controller through the piezoelectric objective locator. The results showed that its frequency tracking bandwidth could reach 255 Hz. Michael et al. [23] used a feedback system consisting of an optical displacement sensor, a 90° phase shifter with feedback gain and a forward path actuator to drive the fast piezoelectric microlens actuator. When the driving frequency was 750 Hz, the minimum switching time of the driver was only 2.5 ms. In order to improve the beam writing quality in confocal differential parallel laser direct writing, Zong et al. [24] designed a leveling focusing servo control system. Though using the differential astigmatism detection method and proportional integral differential (PID) feedback algorithm, the error of light source and external interference is reduced. By means of dynamic focusing, the elongation of piezoelectric ceramics can be guaranteed within the range of focal depth, the discrimination accuracy of the focal position can be nanoscale, and the tilt angle and pitch angle of the focusing platform are around 1 × 10^−5^ rad. Using traditional proportional integral differential control (PID) and fuzzy control, Pan et al. [25] proposed the parallel switching control strategy of the electric objective lens driven by an ultrasonic motor. The predictive control method of macro and micro phase fusion is established to realize the high precision and fast switching control of the objective lens converter. Addditionally, the repeated positioning error of the electric objective lens converter is less than 0.015°. For the drive system of the piezoelectric objective lens in digital confocal microscopy, Chen et al. [26] analyzed the control performance of the fuzzy PID controller and proposed the idea of off-line optimization. The optimized initial parameters are configured in the fuzzy PID control, the system is adjusted online and in real time, and the control system is driven by step positioning, which improves the response speed of the system.

From the above studies, scholars have made many valuable achievements in the field of piezoelectric-driven focusing. Various focusing mechanisms have been developed. However, there are still some problems such as slow response speed, small range of lens movement, small output force and poor stability that exist in focusing mechanisms, which seriously restrict the application of the focusing mechanism in the field of precise focusing and macro focusing. Meanwhile, previous research mainly uses piezoelectric ceramic as the driving source, so the driving force and output displacement of the piezoelectric ceramic plate are small. What is more, the friction mechanism between the stator and rotor of traditional piezoelectric actuation makes the transmission ratio unstable. In order to realize the rapid response, high precision positioning and large displacement focusing of the focusing mechanism, a cylindrical macro focusing mechanism based on piezoelectric drive, is proposed in this paper. In the proposed mechanism model, the stacked piezoelectric actuator is used as the driving source, which can output larger force and displacement than the piezoelectric ceramic. At the same time, the application of movable teeth makes the contact of the transmission mechanism as point contact, which greatly reduces the friction between the transmission mechanisms and makes the transmission ratio adjustable. Therefore, the novel piezoelectric focusing mechanism proposed by the author has important innovations.

Electromechanical coupling characteristic is an important factor of the focusing mechanism, which will affect the driving precision and overall performance of the system. Hence, in this paper, the electromechanical coupling mechanical model and equations of the proposed focusing mechanism are established. The output performance of the focusing mechanism is analyzed as well. What is more, the effect of parameters on output performance is investigated. The research results lay a theoretical foundation for the application of piezoelectric drive in the focusing mechanism.

## 2. Operating Principle of the Piezo-Driven Focusing Mechanism

**Figure 1:**
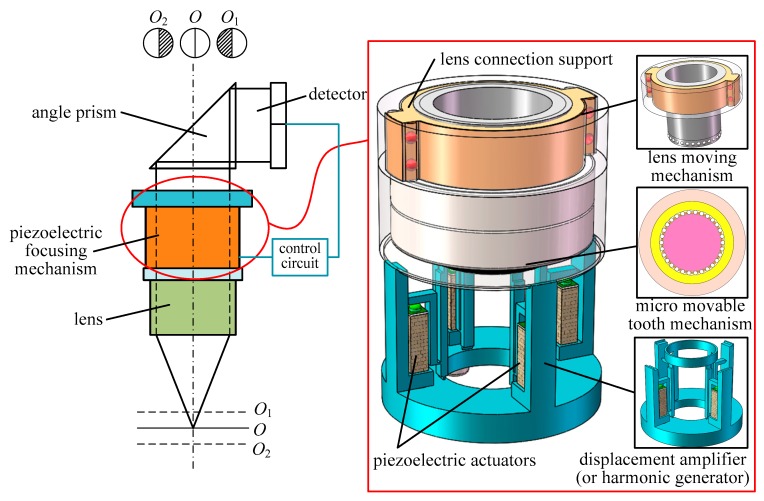
Operating principle of the piezo-driven focusing mechanism.

The operating principle of the proposed piezo-driven focusing mechanism is shown in Figure 1. In the structure diagram, the focusing mechanism consists of the lens moving mechanism, micro movable tooth mechanism and displacement amplifier (or harmonic generator), and integrates piezoelectric drive, harmonic drive, precision gear drive and screw drive. Compared with other piezoelectric focusing mechanisms, the piezoelectric focusing system proposed in this paper has the advantages of large lens displacement, adjustable moving speed, large driving force, integrated drive and deceleration, rolling contact between stator and tooth carrier and between the lens connecting support and guide rail and long service life. Hence, it is suitable for macro focusing of micro target shooting. Through simulation analysis, the output torque of the movable tooth drive system and thrust force of the moving lens are 1.16 Nm and 562.5 N under the driven by four 5 × 5 × 20 mm^3^ piezoelectric actuators. In Ref. [27], the largest output torque of a harmonic piezo-drive motor with eight 5 × 5 × 50 mm^3^ piezoelectric actuators is only 0.75 Nm. Thus, the torque density of the proposed piezoelectric focusing mechanism is more than three times larger than that of the harmonic piezo-drive motor.

During the operation of the focusing system, when the image beam enters the lens focus, it is received by the detector through the reflection of the angle prism for signal calculation and amplification. The signal is transmitted to the macro focusing system, causing the lens to twitch up and down for dynamic defocusing compensation. In Figure 1, Position *O* is the image obtained by focusing, and the light flux in the two quadrants is equal. The positions *O*_1_ and *O*_2_ are in the near-focus and far-focus images, and the light flux in the two quadrants is not equal.

**Figure 2:**
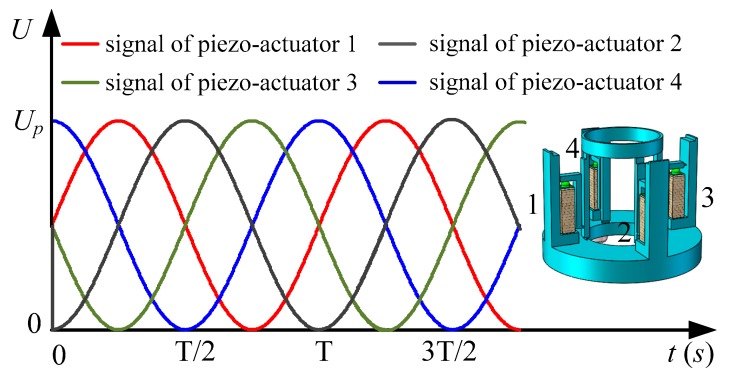
Driving signals of piezoelectric actuators.

The piezoelectric focusing system is driven by four sinusoidal signals whose phases differ by π/2 in turn. As the stacked type piezoelectric actuator is selected in this paper, the actuator is made of several piezoelectric ceramic plates bonded together with fixing glue. For exposed piezoelectric ceramics, once the tension is applied, the adhesive between the ceramic plates breaks. Hence, the piezoelectric ceramic does not resist tension, and the piezoelectric actuators can only apply positive voltages. The driving signals that applied for the piezoelectric actuators are shown in Figure 2. The equations of the driving signals can be expressed by:

(1) $$\left\{ \begin{array}{l} U_{1}\left(t\right)=\frac{1}{2}U_{p}\left[1+\sin\left(2\pi ft\right)\right]\\ U_{2}\left(t\right)=\frac{1}{2}U_{p}\left[1+\sin\left(2\pi ft-\frac{\pi }{2}\right)\right]\\ U_{3}\left(t\right)=\frac{1}{2}U_{p}\left[1+\sin\left(2\pi ft-\pi \right)\right]\\ U_{4}\left(t\right)=\frac{1}{2}U_{p}\left[1+\sin\left(2\pi ft-\frac{3\pi }{2}\right)\right] \end{array}\right.,$$

where *U*_1_, *U*_2_, *U*_3_, *U*_4_ are the voltage that is applied to the piezoelectric actuators; *U_p_* is the peak value of the voltage; *f* is the driving frequency.

**Figure 3:**
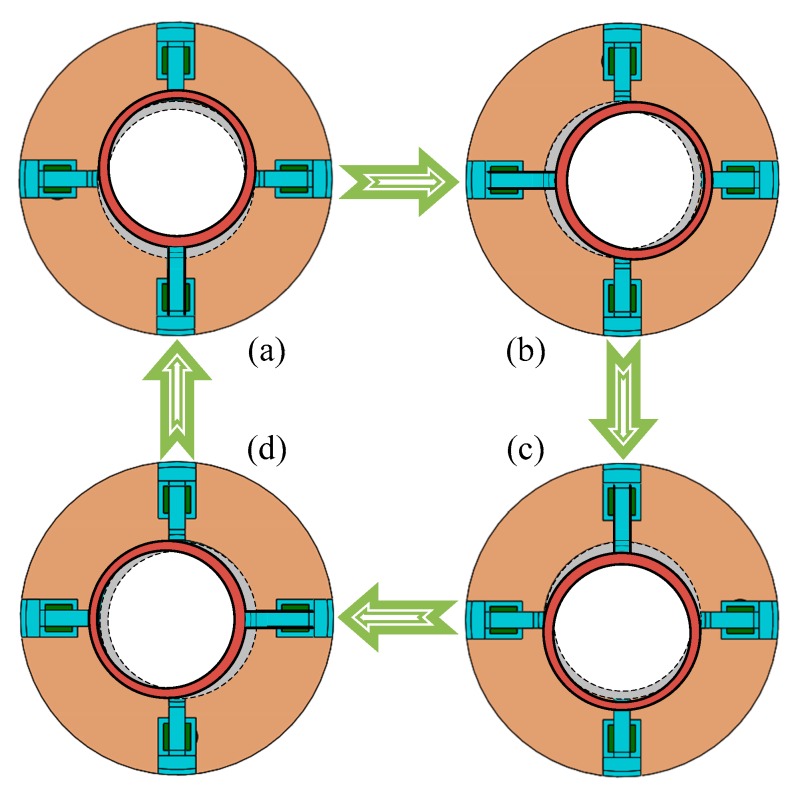
Working principle of the harmonic generator. (**a**) Initial position and 360° position; (**b**) 90° position; (**c**) 180° position; (**d**) 270° position.

Driven by four signals, the harmonic wave is generated on the top surface of the harmonic generator, which is shown in Figure 3. Here, branch Figure 3a–d represent the initial position (or 360° position), 90° position, 180° position and 270° position of harmonic wave, respectively. However, when the order of input signals for piezoelectric actuators changes in Equation (2), the rotational motion turns in the opposite direction.

Hence, the reverse drive signals of piezoelectric actuators can be written as:

(2) $$\left\{ \begin{array}{l} U_{1}\left(t\right)=\frac{1}{2}U_{p}\left[1+\sin\left(2\pi ft\right)\right]\\ U_{2}\left(t\right)=\frac{1}{2}U_{p}\left[1+\sin\left(2\pi ft+\frac{\pi }{2}\right)\right]\\ U_{3}\left(t\right)=\frac{1}{2}U_{p}\left[1+\sin\left(2\pi ft+\pi \right)\right]\\ U_{4}\left(t\right)=\frac{1}{2}U_{p}\left[1+\sin\left(2\pi ft+\frac{3\pi }{2}\right)\right] \end{array}\right..$$

**Figure 4:**
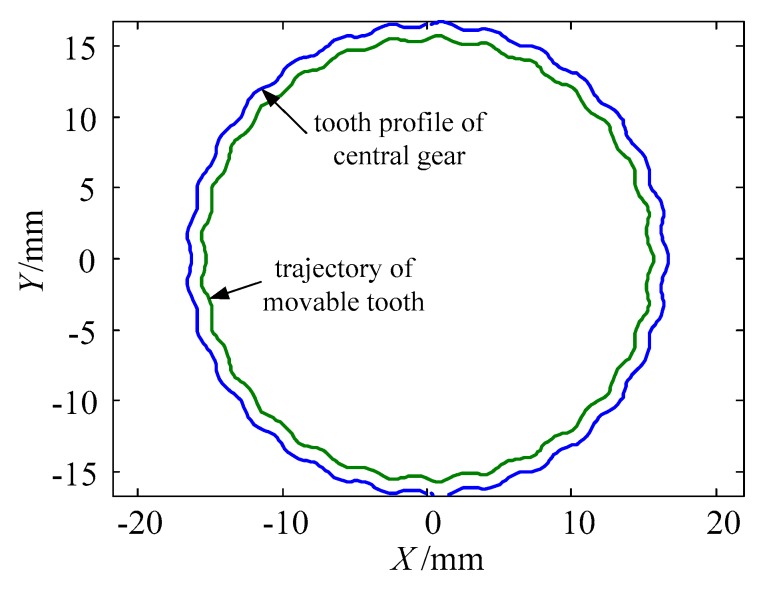
The tooth profile of the central gear and trajectory of the movable tooth.

The harmonic forces push the movable teeth to mesh with the tooth profile of the central gear, and the motion of movable teeth drive the tooth carrier to generate a small angle. The equations and diagram of the tooth profile of central gear and trajectory of movable tooth [28] are presented in Equations (3) and (4) and Figure 4:

(3) $$\left\{ \begin{array}{l} X=b\cos\left(\varphi -\arcsin\left[a\sin\left(\left(i_{cp}-1\right)\varphi \right)/b\right]\right)+a\cos\left(i_{cp}\varphi \right)\pm r_{p}\cos\psi \\ Y=b\sin\left(\varphi -\arcsin\left[a\sin\left(\left(i_{cp}-1\right)\varphi \right)/b\right]\right)+a\sin\left(i_{cp}\varphi \right)\pm r_{p}\sin\psi \end{array}\right.,$$

(4) $$\left\{ \begin{array}{l} x_{p}=\left[a\cos\left[\left(i_{cp}-1\right)\alpha_{j}\right]+\sqrt{b^{2}-a^{2}\sin^{2}\left[\left(i_{cp}-1\right)\alpha_{j}\right]}\right]\sin\alpha_{j}\\ y_{p}=\left[a\cos\left[\left(i_{cp}-1\right)\alpha_{j}\right]+\sqrt{b^{2}-a^{2}\sin^{2}\left[\left(i_{cp}-1\right)\alpha_{j}\right]}\right]\cos\alpha_{j} \end{array}\right.,$$

where φ is the rotational angle of the tooth carrier; *ψ* is the angle between the normal of the moving tooth center and *x* axis; *a* is the wave generator offset; *b* = *r_s_*+*r_p_*, *r_s_* and *r_p_* are the radius of the wave generator and the movable tooth; *i_cp_* is the transmission ratio of the movable tooth system; *α_j_* is the angle between center of the *j*-th movable tooth and geometric center of the center gear.

Under the action of four continuous signals, the tooth carrier realizes continuous rotation. With screw drive, the rotational motion of the tooth carrier is converted into the linear motion of the lens. The proposed piezo-driven focusing mechanism can achieve the function of the large displacement of the lens, adjustable movement speed and low friction force.

## 3. Electrochemical Coupled Models and Equations

The driving forces of the proposed micro piezo-driven focusing mechanism come from four piezoelectric actuators. As the piezoelectric actuator selected here is elongated along the axis and its working mode is *d*_33_ (piezoelectric strain constant), so the working mode of *d*_33_ is adopted in this paper. Except the *d*_33_ mode can meet the work requirements, other modes of piezoelectric actuators can also meet the requirements. Here, only one working mode is utilized for analysis. The output strain of each piezoelectric actuator varies with time, its expression is:

(5) $$\left\{ \begin{array}{l} \varepsilon_{p1}=s_{33}\frac{P_{1}}{S_{p}}+\frac{1}{2h_{p}}d_{33}U_{p}\left[1+\sin\left(2\pi ft\right)\right]\\ \varepsilon_{p2}=s_{33}\frac{P_{2}}{S_{p}}+\frac{1}{2h_{p}}d_{33}U_{p}\left[1+\sin\left(2\pi ft-\frac{\pi }{2}\right)\right]\\ \varepsilon_{p3}=s_{33}\frac{P_{3}}{S_{p}}+\frac{1}{2h_{p}}d_{33}U_{p}\left[1+\sin\left(2\pi ft-\pi \right)\right]\\ \varepsilon_{p4}=s_{33}\frac{P_{4}}{S_{p}}+\frac{1}{2h_{p}}d_{33}U_{p}\left[1+\sin\left(2\pi ft-\frac{3\pi }{2}\right)\right] \end{array}\right.,$$

where *s*_33_ is the elastic flexibility coefficient of the piezoelectric actuator; *P*_1_, *P*_2_, *P*_3_ and *P*_4_ are preloads of the piezoelectric actuators; *S_p_* is the sectional area of the piezoelectric actuator; *h_p_* is thickness of the piezoelectric layer.

Hence, the output forces of piezoelectric actuators can be written as:

(6) $$\left\{ \begin{array}{l} F_{p1}=c_{33}s_{33}P+\frac{1}{2h_{p}}c_{33}d_{33}S_{p}U_{p}\left[1+\sin\left(2\pi ft\right)\right]\\ F_{p2}=c_{33}s_{33}P+\frac{1}{2h_{p}}c_{33}d_{33}S_{p}U_{p}\left[1+\sin\left(2\pi ft-\frac{\pi }{2}\right)\right]\\ F_{p3}=c_{33}s_{33}P+\frac{1}{2h_{p}}c_{33}d_{33}S_{p}U_{p}\left[1+\sin\left(2\pi ft-\pi \right)\right]\\ F_{p4}=c_{33}s_{33}P+\frac{1}{2h_{p}}c_{33}d_{33}S_{p}U_{p}\left[1+\sin\left(2\pi ft-\frac{3\pi }{2}\right)\right] \end{array}\right.,$$

where *c*_33_ is elastic modulus of the piezoelectric actuator.

**Figure 5:**
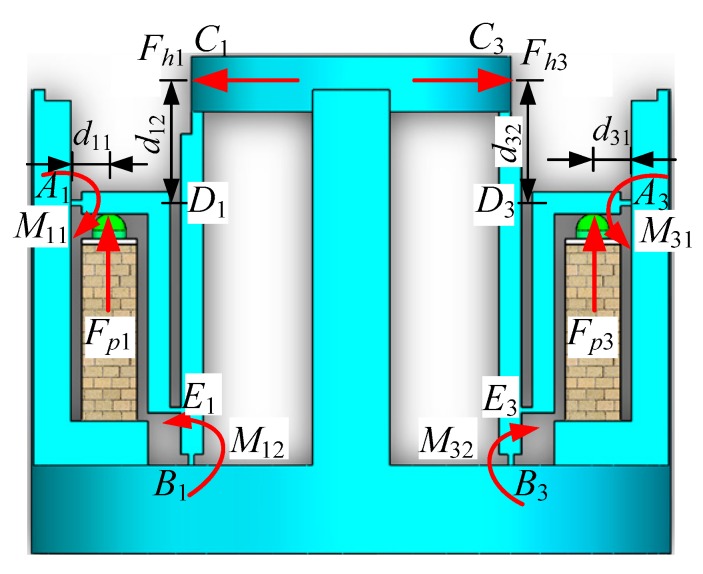
Force diagram of displacement amplification mechanism.

Figure 5 shows the forces of the displacement amplification mechanism, where *F_pi_* is the output force of the piezoelectric actuators, *F_hi_* is the force that applied to the wave generator, *M_i_*_1_ and *M_i_*_2_ are torsional moment of the flexible hinge, *d_i_*_1_ and *d_i_*_2_ are length dimension, and *i* = 1, 2, 3, 4. According to theoretical mechanics, establishing the force balance equation as follows:

(7) $$\left\{ \begin{array}{l} \sum M_{A1}\left(F\right)=0,\;\;\;F_{p1}d_{11}+F_{h1}d_{12}-M_{11}+M_{12}=0\\ \sum M_{A2}\left(F\right)=0,\;\;\;F_{p2}d_{21}+F_{h2}d_{22}-M_{21}+M_{22}=0\\ \sum M_{A3}\left(F\right)=0,\;\;\;F_{p3}d_{31}+F_{h3}d_{32}-M_{31}+M_{32}=0\\ \sum M_{A4}\left(F\right)=0,\;\;\;F_{p4}d_{41}+F_{h4}d_{42}-M_{41}+M_{42}=0 \end{array}\right..$$

Rotational angle of flexible hinge *A_i_* can be expressed by:

(8) $$\theta_{i1}=\arctan\frac{\delta_{np}}{d_{i6}}=\arctan\left[\frac{n}{d_{i6}}\left(s_{33}h_{p}\frac{P}{S_{p}}+d_{33}U_{i}\right)\right],$$

where $\delta_{np}$ is total deformation of the piezoelectric actuator; $d_{i6}$ is length from *A*_1_ to *D*_1_; *n* is the number of the piezoelectric layer for the actuator.

Similarly, the rotational angle of the flexible hinge *B_i_* can be expressed by:

(9) $$\theta_{i2}=\arctan\frac{\delta_{1}}{d_{i4}}=\arctan\left(\frac{1}{d_{i4}}d_{i5}\sin\theta_{i1}\right),$$

where $\delta_{1}$ is the intermediate variable; $d_{i4}$ is the length from *B_i_* to *E_i_*; $d_{i5}$ is the length from *D_i_* to *E_i_*.

According to the torque calculation formula of the flexure hinge [29], the torsional moment of the flexure hinge can be written as:

(10) $$\left\{ \begin{array}{l} M_{i1}=K_{\alpha z}\theta_{i1}\\ M_{i2}=K_{\alpha z}\theta_{i2} \end{array}\right..$$

From Equations (6)–(9), the force that applied to the wave generator can be deduced as follows:

(11) $$F_{hi}=\frac{1}{d_{i2}}\left(K_{\alpha z}\theta_{i1}-K_{\alpha z}\theta_{i2}-c_{33}s_{33}d_{i1}\frac{P_{1}}{S_{p}}-\frac{1}{h_{p}}c_{33}d_{33}d_{i1}U_{i}\left(t\right)\right).$$

**Figure 6:**
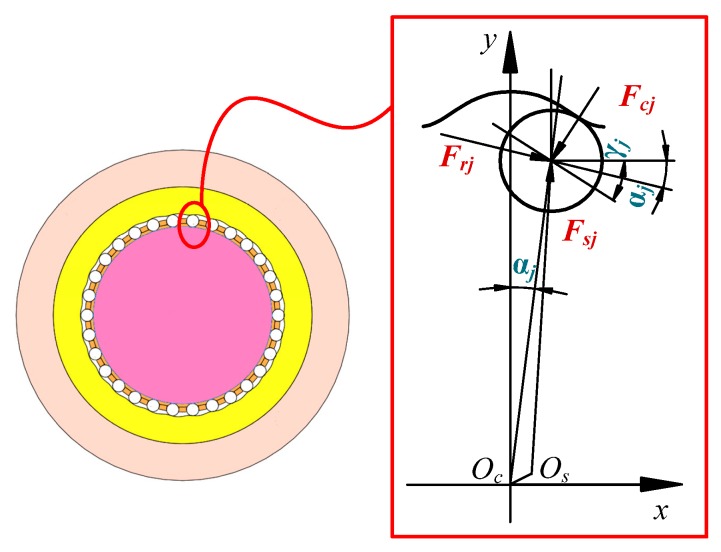
Forces of a selected single movable tooth.

The forces of a selected single movable are shown in Figure 6, where *O_c_* and *O_s_* represent the center of the central gear and harmonic generator, *γ_j_* is the angle between the tangent line of the center track of the movable tooth and the *x*-axis, *F_cj_*, *F_rj_* and *F_sj_* are the forces of the movable tooth that are applied by the central gear, tooth carrier and harmonic generator, respectively. 

The force equilibrium equations of the single movable teeth along *x* and *y* directions can be written as:

(12) $$\left\{ \begin{array}{l} F_{rj}\cos\alpha_{j}+F_{sj}\sin\left[\alpha_{j}-\arcsin\frac{a\sin\left(\left(i-1\right)\alpha_{j}\right)}{b}\right]-F_{cj}\sin\left(-\frac{dy_{p}}{dx_{p}}\right)=0\\ F_{rj}\sin\alpha_{j}-F_{sj}\cos\left[\alpha_{j}-\arcsin\frac{a\sin\left(\left(i-1\right)\alpha_{j}\right)}{b}\right]+F_{cj}\cos\left(-\frac{dy_{p}}{dx_{p}}\right)=0 \end{array}\right..$$

By decomposing the forces of the movable tooth received from the wave generator along *x* and *y* axis, it can be obtained:

(13) $$\left\{ \begin{array}{l} \sum_{j=1}^{n}F_{sj}\sin\left[\varphi_{0}+i_{cp}\varphi +\arcsin\frac{a\sin\left(i_{cp}\alpha_{j}-\alpha_{j}\right)}{b}+\frac{2\pi \left(j-1\right)}{i_{cp}}\right]=F_{h1}-F_{h3}\\ \sum_{j=1}^{n}F_{sj}\cos\left[\varphi_{0}+i_{cp}\varphi +\arcsin\frac{a\sin\left(i_{cp}\alpha_{j}-\alpha_{j}\right)}{b}+\frac{2\pi \left(j-1\right)}{i_{cp}}\right]=F_{h2}-F_{h4} \end{array}\right.,$$

where $\varphi_{0}$ is the initial position angle of the tooth carrier.

Therefore, the output torque of the tooth carrier can be obtained as the following form:

(14) $$T_{r}=\sum_{j=1}^{n}F_{rj}\left[a\cos\left(i_{cp}\alpha_{j}-\alpha_{j}\right)+b\cos\left(\arcsin\frac{a\sin\left(i_{cp}\alpha_{j}-\alpha_{j}\right)}{b}\right)\right].$$

**Figure 7:**
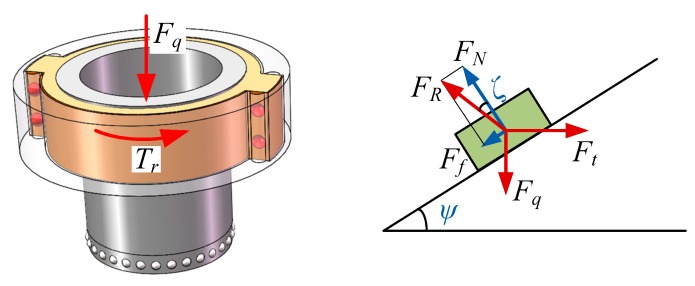
Force diagram of the screw drive mechanism.

When the piezoelectric focusing mechanism works, the output torque of the tooth carrier is transferred to the screw drive mechanism to realize the linear motion of the lens. Figure 7 shows the force diagram of the screw drive mechanism, where *F_q_* and *F_t_* are axial pushing force and horizontal pushing force, supporting force *F_N_* and frictional force *F_f_* constitute the opposite force *F_R_*, *Ψ* and *ζ* are the helix angle and frictional angle of the screw drive.

Establishing the equilibrium equations in the horizontal and vertical directions, it can be obtained:

(15) $$\left\{ \begin{array}{l} F_{t}-F_{N}\sin\psi -F_{f}\cos\psi =0\\ F_{N}\cos\psi -F_{f}\sin\psi -F_{q}=0 \end{array}\right..$$

From Equation (15), it can be deduced that:

(16) $$F_{t}=F_{q}\tan\left(\psi +\arctan\mu_{s}\right),$$

where $\mu_{s}$ is the frictional coefficient.

As horizontal pushing force is provided by the output torque of the tooth carrier, so the relationship between *F_t_* and *T_r_* can be written as:

(17) $$F_{q}=\frac{2T_{r}}{d_{m}\tan\left(\psi +\arctan\mu_{s}\right)},$$

where *d_m_* is the effective diameter of the thread.

Hence, according to above equations, the output characteristic of the proposed piezo-driven focusing mechanism can be acquired. Furthermore, the electromechanical coupling relationship between output force and input voltage is established.

## 4. Results and Discuss 

### 4.1. Output Forces of Piezoelectric Actuator

**Figure 8:**
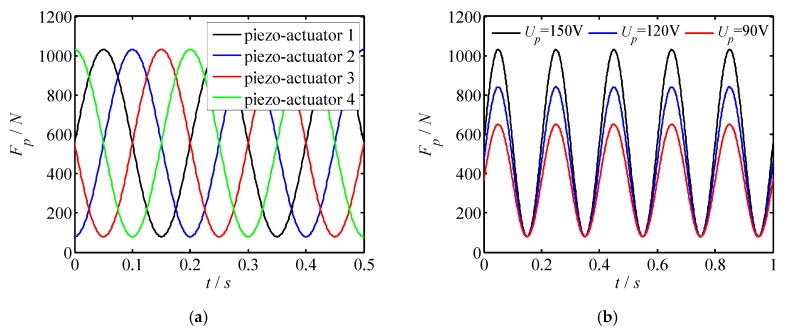

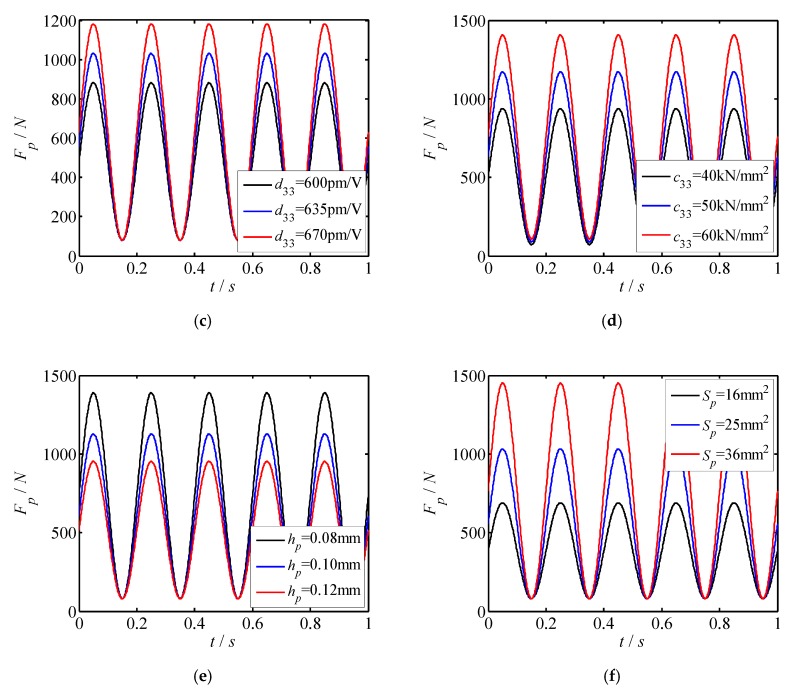
Output forces of the piezoelectric actuator. (**a**) Force changes with different piezo-actuator; (**b**) force changes with *U_p_*; (**c**) force changes with *d*_33_; (**d**) force changes with *c*_33_; (**e**) force changes with *h_p_*; (**f**) force changes with *S_p_*.

For the proposed piezo-driven focusing mechanism, a dimension of 5 × 5 × 20 mm^3^ of the piezoelectric actuator is selected to drive the focusing mechanism. Meanwhile, a 100 N preload is applied to the piezoelectric actuator. Applying those conditions and Equation (6), the output forces of the piezoelectric actuator are investigated as shown in Figure 8, where sub-figure a shows the output forces of the different piezo-actuator, whereas Figure 8b–f represent the changes of output forces with different peak voltage, piezoelectric strain constant, elastic modulus of piezoelectric actuator, thickness of piezoelectric layer and sectional area of piezoelectric actuator. Based on Figure 8, one can draw the conclusions:

(1) The forces acting on the piezoelectric actuator vary sinusoidal with time. The phase difference of the force curve for each piezoelectric actuator is π/2. Besides, when the peak voltage of each piezoelectric actuator is the same, the maximum output force of each piezoelectric actuator is the same as well.

(2) As the driving voltage *U_p_* increases, the output forces of the piezoelectric actuator increase linearly. Therefore, the output force of the piezoelectric actuator can be adjusted by changing the driving voltage.

(3) The variation law of the output force with *d*_33_, *c*_33_ and *S_p_* is similar to that with *U_p_*. The output force is proportional to the change of parameters. 

(4) The output force of the piezoelectric actuator decreases with the thickness of the piezoelectric layer increasing. Hence, a small thickness of the piezoelectric layer benefits to obtain a large output force of the piezoelectric actuator, and then gets better output performance.

### 4.2. Forces of Movable Tooth Drive

**Figure 9:**
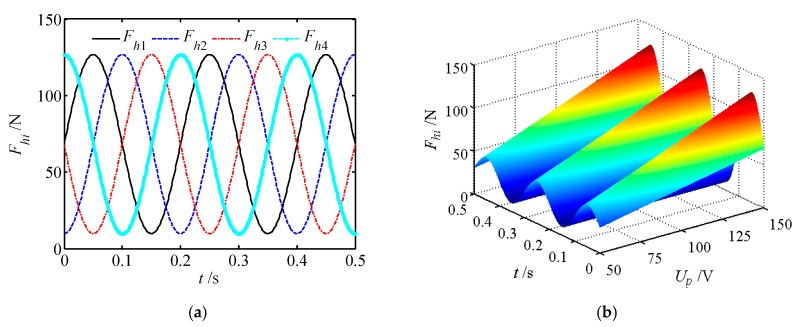
Harmonic force of the wave generator. (**a**) Harmonic under different input signals; (**b**) harmonic force varies with input voltage and time.

According to Equation (11), the harmonic force of the wave generator has been deduced. Using MATLAB simulation software, the harmonic force under different input signals and its variation with voltage and time are studied as shown in Figure 9. From Figure 9, it can be observed that:

(1) The maximum harmonic force is 126.8 N, which is only 1/8 of the output force of the piezoelectric actuator. It can thus be seen that the displacement is amplified 8 times by the action of the displacement amplifier.

(2) What is more, the harmonic force increases linearly with the input voltage at a certain time, whereas it changes sinusoidal with time when the input voltage is constant. 

**Figure 10:**
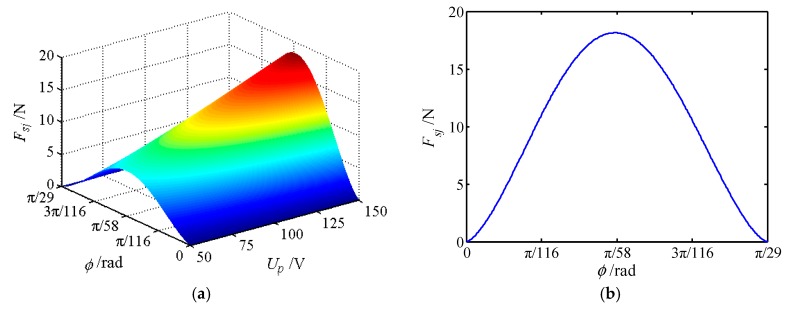
The force of movable tooth *F_sj_*. (**a**) The force varies with rotational angle and voltage; (**b**) force sectional view at *U_p_* = 150 V.

**Figure 11:**
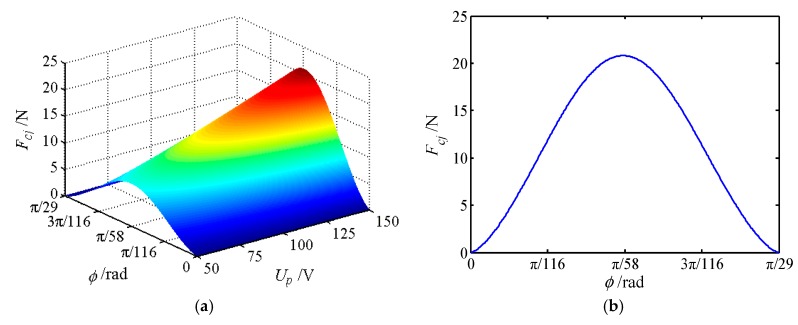
The force of movable tooth *F_cj_*. (**a**) The force varies with rotational angle and voltage; (**b**) force sectional view at *U_p_* = 150 V.

**Figure 12:**
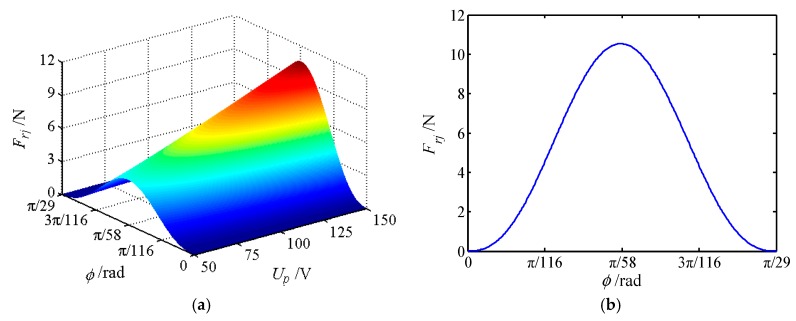
The force of movable tooth *F_rj_*. (**a**) The force varies with rotational angle and voltage; (**b**) force sectional view at *U_p_* = 150 V.

The movable tooth drive system is an important part of the proposed piezoelectric focusing mechanism, and its mechanical characteristics affect the stable output characteristics of the system. Therefore, the force of movable teeth is analyzed in this section, where 30 movable teeth are selected to mesh with 29 teeth of the central gear. Table 1 shows the number of the meshing movable teeth of different positions in a period. Figure 10, Figure 11 and Figure 12 depict the forces of the movable tooth. From Table 1 and Figure 10, Figure 11 and Figure 12, it is known that:

(1) For every π/870 angle that the tooth carrier rotates, one of the movable tooth contacts the tooth tip or root of the central gear. Once again at an angle of π/870, one of the movable teeth falls out of meshing, so π/870 is the dividing point between the movable teeth entering and exiting meshing.

(2) At any given moment, all movable teeth are acted upon by the force from the wave generator *F_sj_*, the central rotation force *F_cj_* and the tooth carrier force *F_rj_*. With the change of the rotating angle of the tooth carrier, the force of each movable tooth will change correspondingly, but its maximum force maintains constant.

(3) The forces *F_sj_*, *F_cj_* and *F_rj_* of a single movable tooth change significantly with the voltage. When the movable gear rotates at an angle of π/58, the force exerted on the movable tooth reaches the maximum value, and the force exerted on the movable tooth is most sensitive to the change of voltage. Forces *F_sj_*, *F_cj_* and *F_rj_* show the tendency of increasing first and then decreasing with the increase of the rotational angle of the tooth carrier.

(4) When the voltage is constant, the peak value of *F_cj_* is the largest, whereas the peak value of *F_sj_* is the smallest. Therefore, the meshing force between the center gear and the movable teeth is the largest. At the same time, the magnitude of force *F_cj_* directly affects the magnitude of *F_rj_*.

### 4.3. Thrust Force of Piezo-Driven Focusing Mechanism

**Figure 13:**
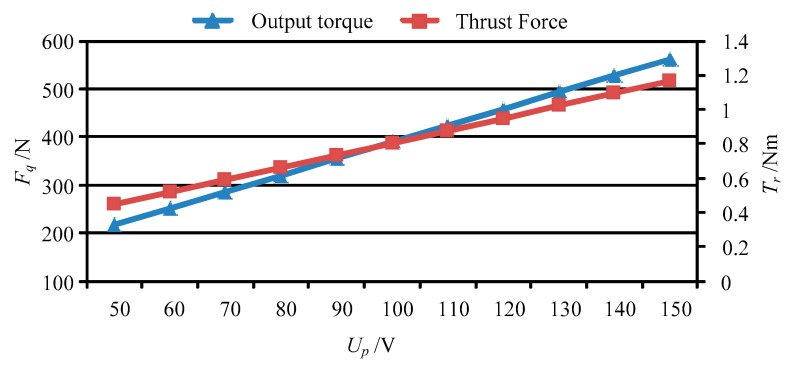
The output torque and thrust force vary with *U_p_*.

**Figure 14:**
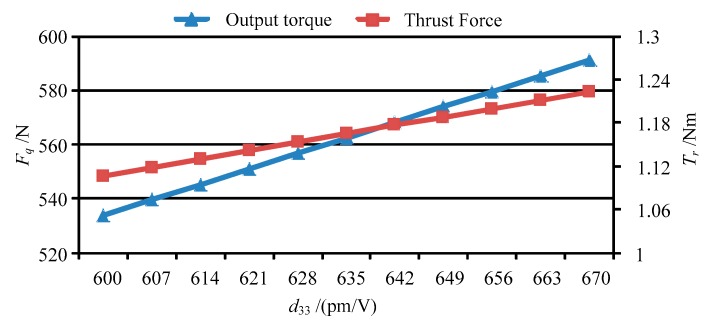
The output torque and thrust force vary with *d*_33_.

**Figure 15:**
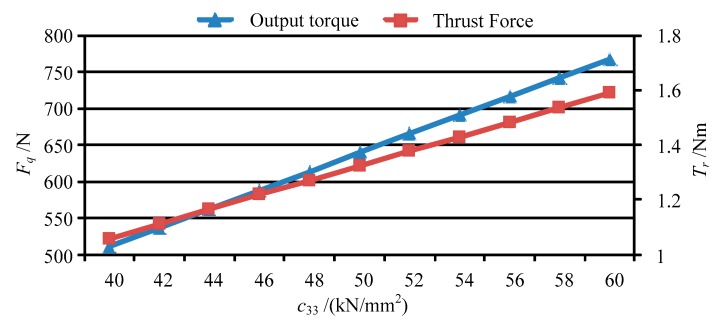
The output torque and thrust force vary with *c*_33_.

**Figure 16:**
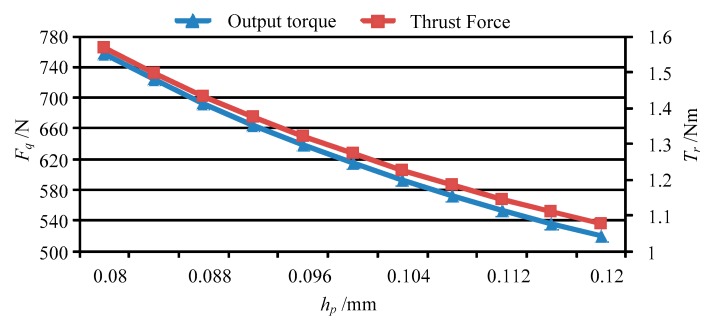
The output torque and thrust force vary with *h_p_*.

**Figure 17:**
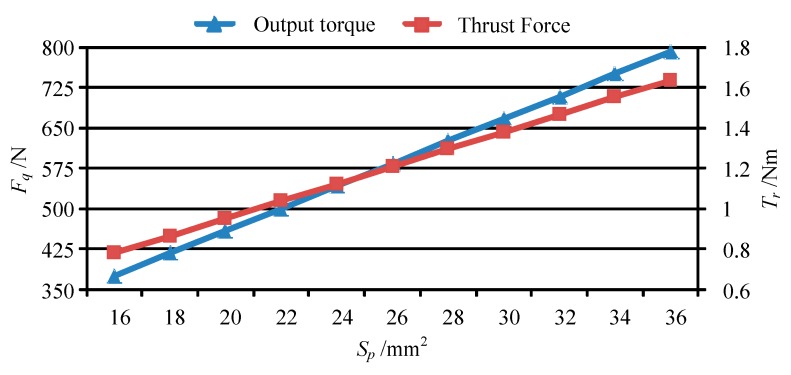
The output torque and thrust force vary with *S_p_*.

Base on the forces of the movable teeth, the torque of the movable tooth drive system can be calculated by using Equation (14), and then the thrust force of the moving lens can be obtained by solving Equation (17). Figure 13 shows the output torque and thrust force vary with peak value of the driving voltage *U_p_*. Figure 14, Figure 15, Figure 16 and Figure 17 depict the output torque and thrust force change with the piezoelectric strain constant *d*_33_, elastic modulus of piezoelectric actuator *c*_33_, thickness of piezoelectric layer *h_p_* and sectional area of piezoelectric actuator *S_p_*. From Figure 13, Figure 14, Figure 15, Figure 16 and Figure 17, it can be concluded that:

(1) With the increase of driving voltage, the output torque of the movable tooth drive system and thrust force of moving lens increase linearly. Therefore, the driving voltage is a decisive factor that affects the output performance of the piezoelectric focusing system.

(2) At *U_p_* = 150 V, the output torque of the movable tooth drive system and thrust force of moving lens are 1.16 Nm and 562.5 N under the driven by four 5 × 5 × 20 mm^3^ piezoelectric actuators. In Ref. [23], the largest output torque of a harmonic piezo-drive motor with eight 5 × 5 × 50 mm^3^ piezoelectric actuators is only 0.75 Nm. Thus, the torque density of the proposed piezoelectric focusing mechanism is more than three times larger than that of the harmonic piezo-drive motor. 

(3) The variation of output torque and thrust force with *d*_33_, *c*_33_ and *S_p_* is similar to that with driving voltage *U_p_*. That is, the output torque and thrust force increase in direct proportion to those parameters. 

(4) As the thickness of the piezoelectric layer *h_p_* increases, the output torque and thrust force decrease nonlinearly. Therefore, the smaller the thickness of the piezoelectric layer, the better the performance of the piezo-driven focusing mechanism.

In short, through theoretical simulation analysis, the proposed micro piezoelectric focusing mechanism has a large driving torque and thrust force, which has an important application prospect in the field lens focusing.

### 4.4. Finite Element Analysis

The finite element analysis (FEA) of the system is carried out by ANSYS software. In the FEA model, the density of the piezoelectric material is 7740 Kg/m^3^, while the stiffness matrix ***c***, dielectric constant matrix ***ε*** and piezoelectric stress constant ***e*** matrix are respectively as follows:

(18) $$c=\left[\begin{array}{cccccc} 12.6 & 7.95 & 8.41 & 0 & 0 & 0\\ & 12.6 & 8.41 & 0 & 0 & 0\\ & & 12.6 & 0 & 0 & 0\\ & & & 2.33 & 0 & 0\\ & & & & 2.3 & 0\\ & & & & & 2.3 \end{array}\right]\times 10^{10}\mathrm{Pa},$$

(19) $$\varepsilon =\left[\begin{array}{ccc} 8.93 & 0 & 0\\ 0 & 8.93 & 0\\ 0 & 0 & 6.92 \end{array}\right]\times 10^{-9}F/m,$$

(20) $$e=\left[\begin{array}{ccc} 0 & 0 & -6.5\\ 0 & 0 & -6.5\\ 0 & 0 & 23.3\\ 0 & 0 & 0\\ 0 & 17 & 0\\ 17 & 0 & 0 \end{array}\right]C/m^{2}.$$

Other parts of the piezo-driven focusing mechanism are made of structural steel. Its material density is 7850 Kg/m^3^, the Young’s modulus and Poisson’s ratio are 2 × 10^11^ Pa and 0.3, respectively. In addition, the compressive yield strength and tensile ultimate strength of this material are 2.5 × 10^8^ Pa and 4.6 × 10^8^ Pa, respectively.

**Figure 18:**
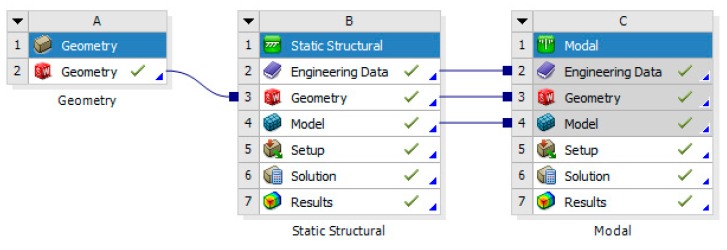
Process of the finite element analysis.

During the finite element analysis, the APDL language of ANSYS is used to program the finite element analysis of the piezoelectric actuator. Then, the analysis results of the piezoelectric actuator are imported into ANSYS Workbench for the finite element analysis of the whole structure of the piezoelectric focusing mechanism. The mesh size was set as 2 mm, and the structure was meshed by free partition type. The finite element analysis process is shown in Figure 18.

**Figure 19:**
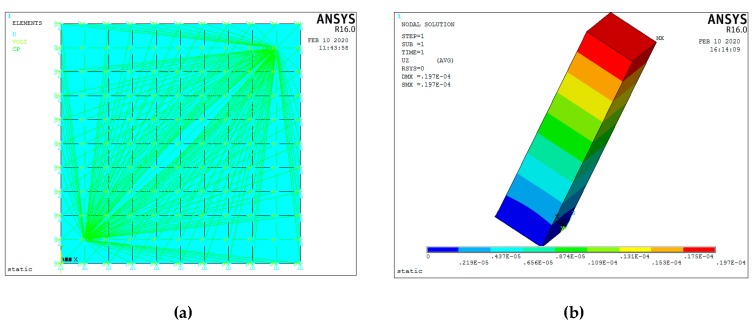

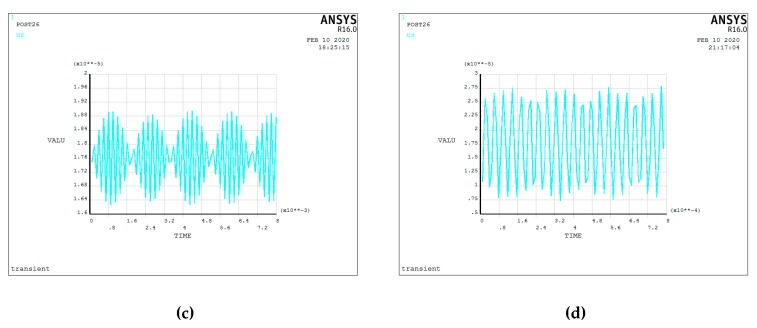
Finite element analysis results of piezoelectric actuator. (**a**) Voltage load applied on the piezoelectric actuator; (**b**) deformation displacement of the piezoelectric actuator; (**c**) transient response at 1000 Hz of the exciting frequency; (**d**) transient response at 5713 Hz of the exciting frequency.

**Figure 20:**
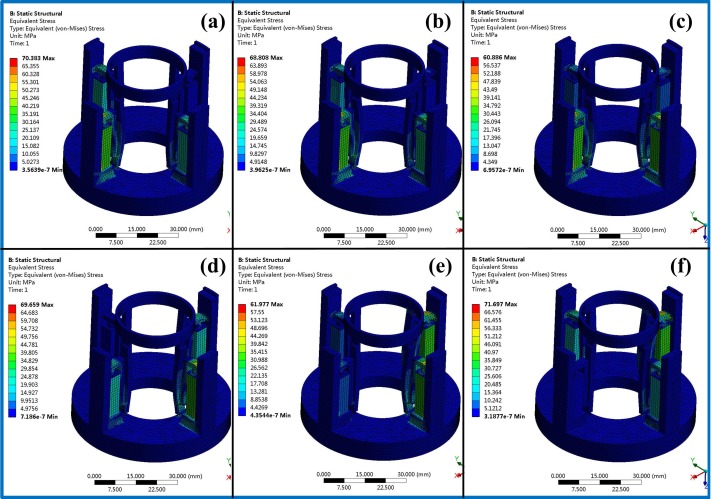
Stress analysis results at different times. (**a**) At *t* = 0 s; (**b**) at *t* = 0.01 s; (**c**) at *t* = 0.025 s; (**d**) at *t* = 0.05 s; (**e**) at *t* = 0.075 s; (**f**) at *t* = 0.1 s.

**Figure 21:**
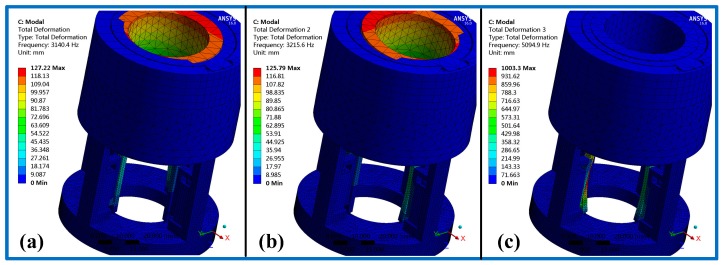
Modal analysis results of the piezoelectric focusing mechanism. (**a**) First-order mode; (**b**) second-order mode; (**c**) third-order mode.

Figure 19 shows the FEA results of the piezoelectric actuator. Its Figure 19a,b give voltage load that applied on the piezoelectric actuator and deformation displacement of the piezoelectric actuator, where a 150 V voltage is applied on it. Figure 19c,d present transient responses at 1000 and 5713 Hz (natural frequency of piezoelectric actuator) of exciting frequency. Figure 20 shows stress analysis results at different times, where a half period time (T/2 in Figure 2) of the exciting signal is selected to illustrate the stress of the displacement magnifying mechanism in different operating conditions. Figure 21 shows the modal analysis results of the piezoelectric focusing mechanism. From Figure 19, Figure 20 and Figure 21, it is known that:

(1) Driven by a voltage of 150 V, the maximum deformation displacement of the piezoelectric actuator is 19.7 μm, which is consistent with the actual output. At the excitation frequency of 1000 Hz, the transient response peak of the piezoelectric actuator is 19 μm. However, this value is 28 μm when the excitation frequency is the natural frequency value 5713 Hz. Thus, the response displacement of the piezoelectric actuator increases greatly when resonance occurs.

(2) According to the stress diagram in Figure 20, the stress of each piezoelectric actuator and magnifying branch changes at different times. The maximum stress occurs at *t* = 0, 0.05 s and 0.1 s. The reason for this is that the voltage of the drive signals of the piezoelectric drivers 1, 2 and 4 reaches the maximum peak at these moments.

(3) The natural frequency values of the first three orders are 3140.4, 3215.6 and 5094.9 Hz. Their corresponding vibration modes are Y swing of the tooth carrier, X swing of the tooth carrier and bending vibration of the displacement amplification mechanism. Since the driving frequency of the piezoelectric focusing mechanism is less than 100 Hz, which is far lower than the natural frequency, thus the resonance has little influence on the system.

In conclusion, the FEM simulation verifies that the response displacement of the piezoelectric actuator is consistent with the actual output, the maximum stress at different moments is within the allowable range, and the operating frequency of the system is lower than the natural frequency.

### 4.5. Principium Experiment

**Figure 22:**
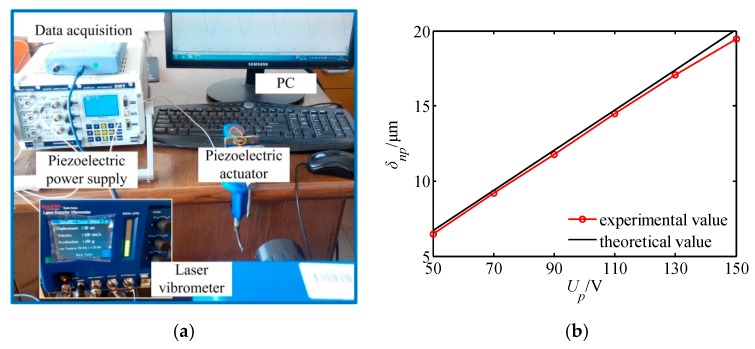
Output displacement test of the piezoelectric actuator. (**a**) Experimental device structure; (**b**) test results.

In order to verify the characteristics of the piezoelectric output, the output displacement of the piezoelectric actuator was tested. Figure 22 shows the output displacement test of the piezoelectric actuator, where the XMT piezoelectric power supply is used as the driving signal and power amplifier, and the OptoMET laser vibrometer is applied to test the displacement of the piezoelectric actuator. From the test results in Figure 22b, it is known that:

The tested displacement is very close to the theoretical displacement, and the maximum error is 2.6% when the voltage is 150 V. The output displacement experiment of the piezoelectric actuator lays a foundation for the development of the prototype.

**Figure 23:**
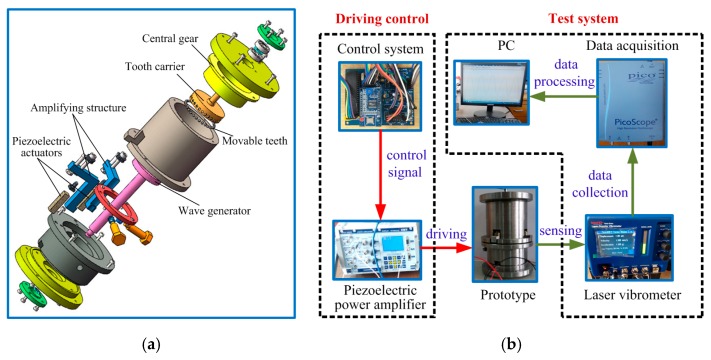
Principium experiment of the piezo-driven focusing mechanism. (**a**) Exploded view of the principium prototype; (**b**) test scheme.

At the same time, in order to verify the feasibility of the design scheme in this paper, the first generation prototype is used to carry out the principium experiment. For the first generation principium prototype, its design principle is the same as that shown in Figure 1. The difference is that the first prototype is driven by two piezoelectric actuators and can only output rotational motion, without a screw drive. The exploded view of the principium prototype and test scheme for the principium experiment are shown in Figure 23, where the control system can output two sinusoidal signals with different phases and positive bias. Table 2 presents the output rotational speed of the principium prototype with different driving voltages. The test results show:

(1) When the peak value of the driving voltage exceeds 100 V, the prototype starts to rotate, and the speed increases slowly with the increase of the voltage. When the peak value of the voltage exceeds 120 V, the speed of the prototype gradually tends to be stable. When the peak value of the voltage is 150 V, the speed of the prototype does not change with the change of the voltage.

(2) When the driving frequency increases, the error between the test speed and the theoretical speed increases. It is because when the driving speed is high, the loss of teeth increases. Therefore, the lower the driving frequency is, the more favorable it is for the system transmission, and the prototype system is more suitable for low-speed work.

In a word, the experimental test verifies the feasibility and reasonability of the piezoelectric drive structure designed in this paper.

### 4.6. Future Work Prospects

Based on the findings of existing studies [30,31] and results of this paper, a static–dynamic hybrid optimization method can be proposed to optimize the performance of the piezoelectric focusing mechanism for future work. The specific steps are as follows:

(1) Based on the results of this paper, a system optimization model will be established to analyze the static performance of single-objective static optimization.

(2) Find out the dynamic objective function, optimize the single objective with dynamic characteristics of the piezoelectric focusing mechanism, and analyze the sensitivity of parameters to dynamic optimization.

(3) Combining static and dynamic optimization goals, establish the static–dynamic multi-objective optimization mathematical model. Compare the difference and relation between the results of multi-objective optimization and single-objective optimization.

Therefore, by using the research results of this paper, the performance optimization of the piezoelectric focusing system can be realized by establishing a multi-objective optimization mathematical model.

## 5. Conclusions

In this paper, a micro piezo-driven focusing mechanism is proposed and its working principle is presented. Using the piezoelectric theory, the movable tooth drive theory and the screw drive theory, the electromechanical coupling mechanical model of the piezoelectric focusing mechanism is established. Through MATLAB simulation, the output characteristics of the piezoelectric focusing mechanism are calculated. The results indicate that:

(1) The driving voltage directly affects the output performance of the piezoelectric focusing mechanism, and the thrust force is proportional to the voltage.

(2) The maximum thrust force of the lens of the piezoelectric focusing mechanism is 562.5 N, and the maximum output torque of the movable tooth drive is 1.16 Nm.

(3) A small thickness of the piezoelectric layer of the piezoelectric actuator benefits to obtain a large output force of the focusing mechanism.

(4) The experiment verifies the displacement output characteristic of the piezoelectric actuator and the feasibility of the proposed piezoelectric drive structure.

These results can be used for improving the output performance of the proposed micro piezo-driven focusing mechanism.

## Figures and Tables

**Table 1:** Number of meshing movable teeth of different positions in a period.

Rotational Angle of Tooth Carrier φ (rad)	Number of Meshing Movable Teeth	Rotational Angle of Tooth Carrier φ (rad)	Number of Meshing Movable Teeth
0–π/870	2–16	π/29–31π/870	17–30, 1
π/870–π/435	2–17	31π/870–16π/435	17–30, 1–2
π/435–π/290	3–17	16π/435–11π/290	18–30, 1–2
π/290–2π/435	3–18	11π/290–17π/435	18–30, 1–3
2π/435–π/174	4–18	17π/435–7π/174	19–30, 1–3
π/174–π/145	4–19	7π/174–6π/145	19–30, 1–4
π/145/–7π/870	5–19	6π/145–37π/870	20–30, 1–4
7π/870–4π/435	5–20	37π/870–19π/435	20-30, 1–5
4π/435–3π/290	6–20	19π/435–39π/870	21–30, 1–5
3π/290–π/87	6–21	39π/870–4π/87	21–30, 1–6
π/87–11π/870	7–21	4π/87–41π/870	22–30, 1–6
11π/870–2π/145	7–22	41π/870–7π/145	22–30, 1–7
2π/145–13π/870	8–22	7π/145–43π/870	23–30, 1–7
13π/870–7π/435	8–23	43π/870–22π/435	23–30, 1–8
7π/435–π/58	9–23	22π/435–3π/58	24–30, 1–8
π/58–8π/435	9–24	3π/58–23π/435	24–30, 1–9
8π/435–17π/870	10–24	23π/435–47π/870	25–30, 1–9
17π/870–3π/145	10–25	47π/870–8π/145	25–30, 1–10
3π/145–19π/870	11–25	8π/145–49π/870	26–30, 1–10
19π/870–2π/87	11–26	49π/870–5π/87	26–30, 1–11
2π/87–7π/290	12–26	5π/87–17π/290	27–30, 1–11
7π/290–11π/435	12–27	17π/290–52π/870	27–30, 1–12
11π/435–23π/870	13–27	52π/870–53π/870	28–30, 1–12
23π/870–4π/145	13–28	53π/870–9π/145	28–30, 1–13
4π/145–5π/174	14–28	9π/145–11π/174	29–30, 1–13
5π/174–13π/435	14–29	11π/174–28π/435	29–30, 1–14
13π/435–9π/290	15–29	28π/435–57π/870	30, 1–14
9π/290–14π/435	15–30	57π/870–π/15	30, 1–15
14π/435–π/30	16–30	π/15–59π/870	1–15
π/30–π/29	16–30, 1	59π/870–2π/29	1–16

**Table 2:** The prototype rotational speed with different driving voltages (r/min).

Voltages	100 V	110 V	120 V	130 V	140 V	150 V
*f* = 0.5 Hz	0	0.492	0.594	0.612	0.625	0.625
*f* = 1 Hz	0	1.017	1.132	1.176	1.188	1.200
